# Expressed Emotion in the Family: A Meta-Analytic Review of Expressed Emotion as a Mechanism of the Transgenerational Transmission of Mental Disorders

**DOI:** 10.3389/fpsyt.2021.721796

**Published:** 2022-02-01

**Authors:** Julia Fahrer, Nathalie Brill, Lisa Marie Dobener, Julia Asbrand, Hanna Christiansen

**Affiliations:** ^1^Department of Clinical Child and Adolescent Psychology, Faculty of Psychology, Philipps University Marburg, Marburg, Germany; ^2^Department of Special Needs Educational and Clinical Child and Adolescent Psychology, Faculty of Psychology and Sports Science, Justus-Liebig-University Gießen, Gießen, Germany; ^3^Institute of Psychology, Humboldt-Universität zu Berlin, Berlin, Germany

**Keywords:** Expressed Emotion, children of parents with a mental illness, COPMI, transgenerational transmission, mental disorders, risk factor, parent child interaction

## Abstract

**Background:**

High Expressed Emotion (HEE) has been identified as a risk factor for the exacerbation and course of mental illness. EE has been investigated as a caregiver's response to an offspring's problem behavior and pathology. The present meta-analysis regards EE from a transgenerational perspective and as one mechanism that might explain the transgenerational transmission of mental disorders.

**Method:**

We identified a total of 13 studies relying on 16 independent samples of parent-child dyads of parents with a mental illness and healthy controls; these were included in our analysis. Results were synthesized into one effect size per sample; meta-regression on additional effects of parental diagnostic category, child mental illness, and child age were also applied.

**Results:**

Parents with a mental illness are classified as HEE significantly more often. Effects were established for high criticism, albeit of small size (OR = 1.45), although they become stronger whenever offspring exhibit mental illness themselves (OR = 2.82).

**Conclusion:**

The current study highlights the dearth of studies on EE in families in which a parent has a mental illness and its effects on their children. Our findings highlight EE as a potential mechanism for attributing the transgenerational transmission of mental disorders, especially for the EE-variable criticism, indicating dysfunctional parent-child interactions.

**Systematic Review Registration:**

http://www.crd.york.ac.uk/PROSPERO/display_record.php?ID=CRD42019117609, identifier: CRD42019117609.

## Introduction

Worldwide, about 12.1–38.5% of children and adolescents are living with a parent who experiences a Mental illness^1^ (1–4). A parent's mental illness is a powerful risk factor (OR 2.4) for their offspring to develop mental health problems (5), and about one third of the children of parents with a mental illness experience serious mental illness later in life (6). Many studies have shown adverse outcomes in children of parents with a mental illness, including children's attachment problems, internalizing, and externalizing behavior problems as well as social, cognitive, physical, and mental illness (6–10).

**Figure 1:**
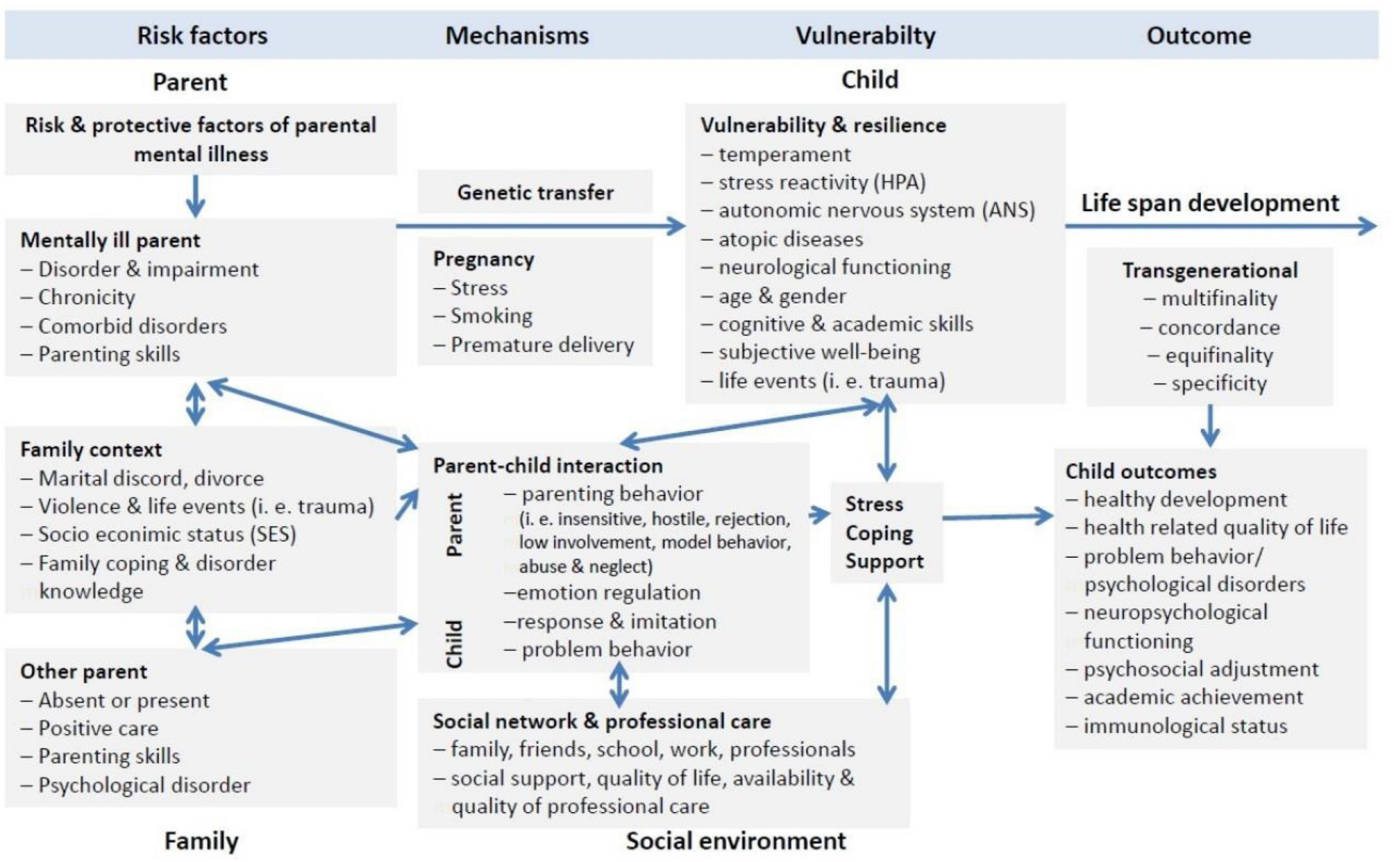
Model of transgenerational transmission of mental illness adapted from Hosman et al. (9) and Christiansen et al. (11).

The Transgenerational Transmission of Mental Disorders system was developed and advanced to provide a comprehensive model to explain such transgenerational transmission of disorders in children of parents with a mental illness (9). This model identifies four major domains (i.e., 1. parent, 2. family, 3. child, 4. social environment) that interact with their respective systems and are influenced by five transmission mechanisms (i.e., 1. genetics, 2. prenatal factors, 3. parent-child-interaction, 4. Family, and 5. social factors) (11). Child development over its life span is considered, as are the concepts of multi- and equifinality, concordance, and specificity (9). Specifically parent-child-interaction is considered to be a core mechanism contributing to the heightened risk of children of parents with a mental illness for developing a serious mental illness (9, 12, 13) (see Figure 1).

### Parent-Child-Interaction

Parenting behaviors are influenced by parental psychopathology, attitudes, attributional styles as well as the child's characteristics on a dyadic level (14, 15). Interaction patterns in families of parents with a mental illness are characterized by elevated rates of insensitive, for example, intrusive, hostile, and critical parental behaviors, the lack of parental warmth and the shortage of acknowledgment of children's developmental, emotional, and attachment needs (7, 10). However, different mental disorders have a different impact on parental behavior and its manifestation (6). Disrupted parental behavior poses a risk for child development and usually is studied with restriction to one diagnosis, but not comparing multiple diagnosis within one study. For instance, mothers with postnatal depression (for example, lower amount of affectionate touch, sensitivity, reciprocity) show a different relational behavioral profile when interacting with their child than mothers with anxiety disorders (e.g., maternal overstimulation of the child, high maternal intrusiveness, parent led interaction) (16). Traditionally, parent, child and dyadic behavior is assessed with standardized, videotaped paradigms and coded with behavior observation schemes (17). Besides these standardized behavior observation schemes, Expressed Emotion (EE) appears to be indicative of dysfunctional parent-child interactions (18), and thus an assessment of interest in this context.

Over the past five decades, EE has been a concept of interest in the field of family relations, and is regarded as an indirect measure of the emotional family climate. Developed for parents of adult patients with schizophrenia, EE was identified to play an important role in the course and relapse of mental illness (19). After controlling for patient variables, such as severe behavior or work impairment, EE still appears to be indicative of negative interactions within a family (20). EE reflects a person's affective attitude toward a close relative and is believed to play an important role in the development and perpetuation of mental disorders in offspring (20–22). It is differentiated in High (HEE) or Low Expressed Emotion (LEE). HEE reflects a high amount of criticism, hostility (CRIT), and/or Emotional Overinvolvement (EOI), whereas LEE is characterized by positive or neutral remarks, low hostility, criticism or emotional overinvolvement toward a close relative and in relationships within families (22, 23). High CRIT levels are linked to negative parental behaviors, such as more parental antagonism, harshness, negativity, and disgust. Low levels of CRIT are associated with more responsive and supportive parenting behavior (18). A current meta-analysis by Rea et al. (24) on the Five-minute Speech Sample (FMSS) in children and youths with internalizing and externalizing symptomatology supports the overall validity of HEE especially with CRIT in the context of child and adolescent health, while the EOI measure appears less robust in such contexts. Nevertheless, the analysis identifies a very small but significant effect between parental EOI and child internalizing symptoms however this result should be interpreted with caution, as the authors point out, that the effect may be caused by specific EOI criteria rather than the construct as a whole (25) and EOI may require more clarification and adaptation (24). Therefore, HEE, and predominantly CRIT, can be perceived as one mechanism of disrupted parent-child interactions in the Transgenerational Transmission of Mental Disorders. EE can be assessed via the Camberwell Family Interview (21, 26, CFI), the FMSS; (27), the Preschool Five-Minute Speech Sample (28, PFMSS), and questionnaires such as the Family Attitude Scale (29, FAS) or Family Questionnaire (30, FQ). Despite the incorporation of hostility in the CFI, it is not captured within the FMSS coding guidelines, as it shows a great overlap with CRIT (31) and does not appear with enough frequency (27). Therefore, the hostility rating is not included in the present analysis and this paper focuses on CRIT and EOI.

### Parents With a Mental Illness and EE

EE traditionally was developed to assess caregiver's attitudes on adult patients with schizophrenia. Attributions that perceive the cause of problem behavior as internally controllable by the patient/offspring result in more negative emotional responses (32, 33), and there is a strong link between attribution theory and EE. There appears to be an attribution-negative affect link in HEE relatives linking hostility and CRIT to negative affect (15). CRIT is assumed to be a correlate of the typical cognitive and attributional style of mothers with depressive disorders (8) and has been identified as a possible moderator of the association between maternal depression and a child's internalizing and externalizing symptoms (34, 35). In contrast to this, fathers with depression do not present with higher levels of CRIT, but do make fewer warm and positive remarks than healthy controls (36), although this is no component of the traditional HEE component. Based on such finding, a sex difference regarding CRIT and parental depression might be assumed. Mothers and fathers with a mental illness are five to nine times more likely to be classified as HEE than parents without any mental health condition (37). Parental EE status seems to be relatively stable over time (38) creating a challenge for vulnerable, genetically predisposed children, and it therefore has the potential to promote a self-perpetuating cycle of children's problem behavior and HEE within a family (39). Given that parents with a mental illness may have been exposed to parental HEE themselves, they may be prone to reacting more negatively, hostilely, and improperly when facing their children's challenges and problem behavior and therefore exhibit HEE, especially CRIT (40, 41). While there are indications that HEE is more prevalent in families in which a parent has a mental illness, predominantly depression (42, 43), generalizable evidence is lacking. Previous research on EE in the field of child and adolescent psychology has been focusing on clinically referred children (44) or the emotional family climate within families of children with internalizing and externalizing symptoms, respectively (24).

### EE in the Field of Child Psychology

In the field of clinical child and adolescent psychology, HEE is regarded as an indicator of the quality of the parent-child-relationship (45). As EE reflects parental attitudes (38, 39) and HEE is a correlate of negative parental behaviors (18), it is not clear whether negative parental attitudes result in more negative parental behaviors, or vice versa. Parental HEE is linked to difficult child temperament (38, 46), and is a correlate of disruptive attachment patterns (47). Parental EE is considered a stable predictor for the course of mental illness and treatment response in children and adolescents (37, 44, 48–50). Low levels of warmth, increased hostility and critical comments have been associated with children's behavioral problems (44, 45, 51–53) and antisocial behavior (49). Parents of children with one axis I diagnosis are significantly more likely to be classified as HEE than parents of healthy controls (37, 54, 55). They appear even more critical when children carry an additional axis I diagnosis to depression (56, 57). Moreover, HEE has been positively identified in predicting the onset of attention deficit hyperactivity disorder (ADHD) (38), comorbid oppositional defiant disorder (ODD) (58), the clinical course of childhood anxiety, bipolar and depressive disorders (49, 59, 60), as well as the treatment response of adolescents suffering from eating disorders (61–63). Neither an offspring's sex nor a family's socio-economic status (SES) are associated with the parental EE status (37, 64) and the assessment of parental psychopathology or burden has achieved little attention when studying EE and child development. Therefore, EE has been and remains a risk factor of interest in the field of clinical psychology and a potential mechanism for explaining the transgenerational transmission of mental disorders. While most articles claim EE to be stable over time (38, 65–67), other methods (e.g., clinical interviews) show some evidence that EE might be somewhat able to change (68). This warrants further research because it might be an interaction with changes in offspring's behavior due to developmental steps, especially at the time before school entry.

### Aims

Although extensive research has been carried out on EE and offspring's psychopathology, comparatively little is known about EE in families in which a parent had a mental illness. The following meta-analysis aimed to investigate HEE as a typical cognitive and affective style of parents with a mental illness.

Moreover, we focused on children of parents with a mental illness and parental EE, assuming EE to be a transgenerational mechanism facilitating the development of mental illness in children of parents with a mental illness (39). The current study aimed to contribute to the current literature by first presenting a comprehensive, quantitative report on the prevalence of HEE in families in which a parent has a mental illness and control families. Secondly, we aimed to identify moderators of the relationship between parental psychopathology and HEE to compute a meta-regression. Therefore, we predicted that parents of younger children tend to show less HEE. Parental diagnosis, sex, and presence of youth psychopathology may account for additional effects on the parental EE status.

## Methods

### Data Sources and Searches

**Figure 2:**
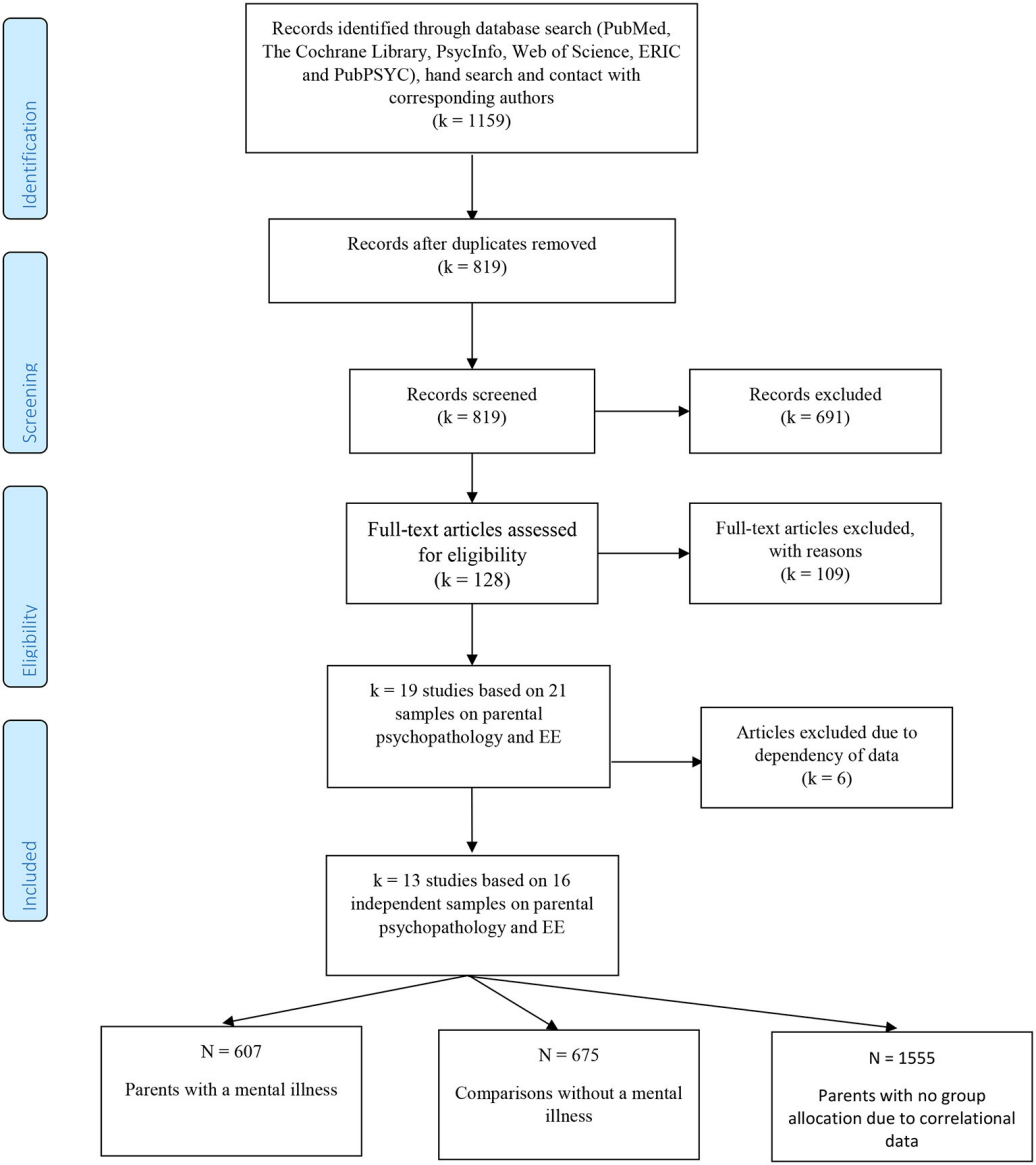
Study flowchart.

This meta-analysis was performed according to the “Preferred Reporting Items for Systematic Reviews and Meta-Analyses” (PRISMA) statement (69). We conducted our search in the following databases: PubMed, The Cochrane Library, PsycInfo, Web of Science, ERIC, and PubPSYC (see Appendix A for search terms). We restricted our search to experimental and observational studies and meta-analyses published in the English or German language until November 2021. Search criteria included parents of minor children as the population addressed, all mental disorders, and the standardized assessment and report of EE or employing a shared measure of EE (see Appendix A). The review protocol is registered on PROSPERO (http://www.crd.york.ac.uk/PROSPERO/display_record.php?ID=CRD42019117609; registration number CRD42019117609). In total, 1,159 studies were identified. Figure 2 shows the flowchart with all study extraction stages.

### Inclusion Criteria

Abstracts of all studies identified from the initial search were screened based on specific inclusion criteria. Studies were included if they reported (a) an experimental or observational design based on (b) a sample of parents of children aged 18 years or younger, (c) a standardized assessment or screening of parental psychopathology and a diagnosis according to DSM or ICD, and (d) a valid measure of parental EE such as the CFI (21, 26), FMSS (27, 70), PFMSS (28), FAS (29), or FQ (30), or results of common parental EE scales, such as HEE or LEE, EOI, or Criticism.

For our meta-analysis of parental psychopathology and expressed emotion, parents had to be classified as the index patient to ensure an estimation of predictive effects. Control conditions had to be no intervention or an internal comparison in case of cohort studies. Furthermore, parents and children had to be free of pervasive developmental disorders to avoid adverse factors caused by these. Studies comparing an active intervention with medication (i.e., psychotherapy vs. medication only) were excluded. All studies included had to provide sufficient information to calculate effect sizes (e.g., means and SDs, *T*-tests for independent samples, n per subgroup, r, Odds Ratio).

### Study Selection

Studies, titles, and abstracts were screened by one reviewer (JF) and relevant studies were extracted that matched our aim and inclusion and exclusion criteria specified for this review. In this respect, a systematic two-stage screening process to identify relevant studies was applied and two authors (JF and LMD) carried out full text screening independently. Discrepancies were resolved through discussion with a third author (HC).

### Study Characteristics/Data Collection Process

Each study was coded on several different domains including publication year, country, primary study aims, study design (e.g., control group), setting, recruitment method, length of follow up, inclusion and exclusion criteria, study participants (i.e., age, sex, diagnosis of parent and children) characteristics of the applied diagnostic instruments or screenings, assessment measure and report of EE status, and the blinding of EE raters. Furthermore, parental diagnostic category was dummy coded, differentiating on a superordinate level of diagnosis, for example, depressive disorders, anxiety disorders. Since information on the children's diagnoses was lacking, only the presence or absence of a diagnosed mental illness was coded. As studies reported inconsistent EE outcome categories, presence or absence of HEE/LEE, CRIT, and EOI was coded in the first step. As CRIT was reported predominantly and information on EOI was lacking, only data on CRIT were extracted. Presence or absence of statistical parameters (M, SD, SE, CI, correlation and regression coefficient, β, χ^2^, B) and N per subgroup was coded. As not all studies reported children‘s mean age but rather age ranges, we coded age categories as well (infants ≤ 20 months, preschoolers > 20 months and ≤ 6 years, school age children > 6 years and ≤ 12 years, and adolescents > 12 years and ≤ 18 years). When defining the age range for the school aged children, we followed the typical age of school entry in Germany even though this might deviate from school entry ages in other countries, as that is where the authors of the study are based.

A subset of study data was extracted by two raters independently, and inter-rater reliability was calculated for each variable. Inter-rater agreement for the coded study characteristics was *k* = 1.00 indicating perfect agreement, except for the variable type of comparison with *k* = 0.43 that resulted in moderate agreement (71).

Interrater-agreement for the coded moderator variables was between *k* = 0.57–1.00, indicating substantial agreement. Study quality was coded with *the Newcastle Ottawa rating scale for observational studies* NOS (72) by two independent raters (JF and NB). The coders completed a standardized form for each study independently to compute inter-rater reliability. An overall quality score was calculated by adding up all the criteria resulting in a maximum score of 9 for each study described in the Supplementary Material. Inter-rater agreement was *k* = 0.49 indicating moderate agreement (71). This is in line with results from recent research, indicating poor to medium inter-rater agreements on the NOS rating scale (73).

### Effect Size Calculation

For the meta-analysis reporting on parental psychopathology and EE, correlational data were transformed into Fisher's *z*, studies reporting continuous data/means and standard deviation were transformed into Cohen's d and binary/dichotomized data into log odds ratios (74). Conversion of Effect sizes into Log Odds Ratios and variance was performed with esc Version 0.5.0 for R (version 3.6.1). Log Odds Ratio was used as the common index for meta-analysis to analyze the odds of being classified as HEE in groups of parents with a mental illness and healthy controls, and transformed back into Odds Ratios (OR) afterwards for improved intelligibility (74). In the case of studies comparing parents with a history of mental illness with current mental illness and to healthy controls, we chose the group currently suffering from symptoms. In case of different articles reporting on the same study but referring to a subsample's different sample sizes (e.g., the subsample of mothers or fathers with incomprehensible dropouts), the paper with the highest quality rating was chosen for meta-analysis to ensure one effect size per sample entering the analysis (75).

Meta-analysis was performed with Metafor Version 2.1-0 for R (Version 3.6.1) using the random effect model (REM) with DerSimonian-Laird method estimator for effect variance τ^2^ (76, 77). Furthermore, heterogeneity of the estimated true effect was calculated using Cochran's *Q*-test for homogeneity and the *I*^2^-statistic (78). Moderator analysis followed heterogeneous effects to identify influencing variables. For continuous variables, moderator analysis was performed with metafor using univariate metaregression models (MEM). Categorical moderator analyses were performed with metafor, recoding categorical variables into dichotomous dummy variables. For all estimated true effects, sensitivity analyses were performed using fixed effect models (FEM) as implemented in Metafor to examine biases due to the choice of the meta-analytic model. Additionally, the influence of potential outliers was examined by using studentized deleted residuals (79). Furthermore, publication bias was examined by funnel plot inspection and test of asymmetry with a rank correlation (80) and regression test (81).

## Results

Data on 16 independent comparisons (parents with a mental illness vs. without) derived from 13 studies with a total of *N* = 2815 parents (*n* = 2,254 mothers, *n* = 561 fathers) were available for our analyses. The sample consisted of 607 parents with a diagnosis of a mental disorder, 675 healthy parents. Further, 1,533 parents could not be assigned to the group of parents with or without a mental illness because only correlational data on the association between EE and parental mental illness was reported from both parents with and without a mental illness within the same group (see Table 1). Nevertheless, OR were computed for the correlation based data. Studies were conducted in the USA, UK, and Australia.

**Table 1:** Characteristics of parental psychopathology and EE studies with means, SDs, percentages.

Parental disorder	Maternal depression: 11 studies Maternal and paternal depression: 2 studies Anxiety disorder: 1 study Affective disorder, not further specified: 1 study
Family composition	80% mothers-child dyad 20% father-child dyad
Parental age	36.68 years (SD = 6.68 years)
Children's age	≤ 20 months (infants): 3 studies ≤ 6 years (pre-schoolers): 3 samples ≤ 12 years (school age kids): 7 samples ≤ 18 years (adolescents): 3 samples
Children's sex	54.7% female
Study design	Observational: 0 Longitudinal studies: 9 Experimental: 0 Controlled trials: 3 Randomized controlled trial: 1
Assessment setting	Clinic: 1 study (8%) Home: 4 studies (31%) Home and centre based: 3 studies (23%) Not reported: 5 studies (38%)
EE assessment tool	FMSS: 10 studies (79%) Preschool Five Minute Speech Sample: 1 study: (7 %) Camberwell Family Interview: 1 study (7%) Family Attitude Scale: 1 study (7%)
Grouping	Parents with a diagnosis of mental illness: *N* = 607 (*n* = 184 male, *n* = 423 female) Parents without any diagnosis of a mental illness: *N* = 675 (*n* = 219 male, *n* = 456 female) Group Membership n.A. due to correlational data: 1,533

For details on the studies as well as parental disorders, see Table 2. Study quality (Table 2; Supplementary Material) was generally medium with 6.25 on the NOS (min. 2, max. 9). Studies predominantly reported CRIT and lacked information on EOI. Because of this, the future analysis only refers to data on the CRIT specification of the HEE construct. Across 13 studies with k = 16 independent samples and unique effects, overall parental psychopathology was positively associated with CRIT (${\hat{\mu }}_{0}$ =1.34 [95% CI = 1.01–1.77] *p* <0.05). Cochran's *Q*-Test suggests variability among true effects (Q = 35.28, df = 15, *p* = 0.022). The variance in the true effect is estimated to be τ^2^ = 0.15. The amount of total variability between the observed effect sizes due to heterogeneity is estimated to be *I*^2^ = 57.49%, and was “moderate” (78).

**Table 2:** Studies included for meta-analysis with the dependent variable parental EE.

**Study**	**Country**	**Disorder studied in parents**	**Clinical assessment tool**	**Setting**	**Female rate**	**Parent's age in years (M)**	**Child's age (M)**	**EE assessment**	**Study quality**	***N***	**OR**
Psychogiou et al. (82)	UK	Depression	SCID, PHQ-9	Home	48%		3.9 years (SD = 0.8)	PFMSS	9	302	
					Mothers	36				144	1.55
					Fathers	39				158	1.34
Mellick et al. (83)	USA	Depression	SCID-I	N.A.	100%	40	10–16 months	FAS	6	81	2.08
Gravener Davis (84)	USA	Depression	DIS-IV, BDI	Home and centre based	100%	N.A.	24 months	FMSS	4	205	1.73
Gravener et al. (34)	USA	Depression	DIS-IV, BDI	Home and centre based	100%	31.68	20 months	FMSS	8	198	1.87
Burkhouse et al. (85)	USA	Depression	SADS-L, BDI-II	N.A.	100%	38.56	9.97 years	FMSS	7	100	0.87
Tompson et al. (86)	USA	Depression	SCID, BDI	Home and centre based	100%	42.2	8–12 years	FMSS	0	171	2.82
Gibb et al. (87)	USA	Depression, anxiety disorder	SADS-L, BDI	N.A.	100%	38.56	9.97 years (SD = 1.32)	FMSS	6	100	0.86
Netsi (36)	UK	Depression	SCID-I, EPDS	Home	50%	33.11	12 months	FMSS	7	103	0.70
Frye and Garber (42)	USA	Depression	SCID	N.A.	100%	38.56	11.88 years (SD = 0.55)	FMSS	7	194	2.36
Nelson et al. (88)	USA	Depression	SCID-IV, BDI	Home	100%	41	15.2 years	FMSS	9	739	1.31
Brennan et al. (89)	AUS	Depression	SCID	Home	0%	25.58 (at time of birth)	15.2 years	FMSS	8	300	0.68
Hirshfeld et al. (90)	USA	Depression, anxiety disorder	DIS	Clinic	100%	N.A.	11 years	FMSS	8	70	3.00
Schwartz et al. (91)	USA	Affective disorders	SADS-L	N.A.	100%	N.A.		CFI	4	252	
							1–9 years			25	4.8
							10–14 years			104	0.06
							15–19 years			123	1.25

*Annotation: Studies are ordered by publication year with the most recent being at the top. N.A., not available; SCID, Structured Clinical Interview for DSM Disorders; SCID-IV, Structured Clinical Interview for DSM IV Disorders; PHQ-9, Patient Health Questionnaire-9; DIS-IV, Diagnostic Interview Schedule for DSM-IV; BDI, Beck's Depression Inventory; BDI-II, Beck's Depression Inventory Revision; SADS-L, Schedule for Affective Disorders and Schizophrenia- Lifetime version; EPDS, Edinburgh Postnatal Depression Scale; EE Assessment, Expressed Emotion Assessment; FMSS, Five Minute Speech Sample; PFMSS, Preschool Five Minute Speech Sample; CFI, Camberwell Family Interview; FAS, Family Attitude Scale; N, total number of participants; OR, Odds Ratio*.

### Sensitivity Analyses

**Figure 3:**
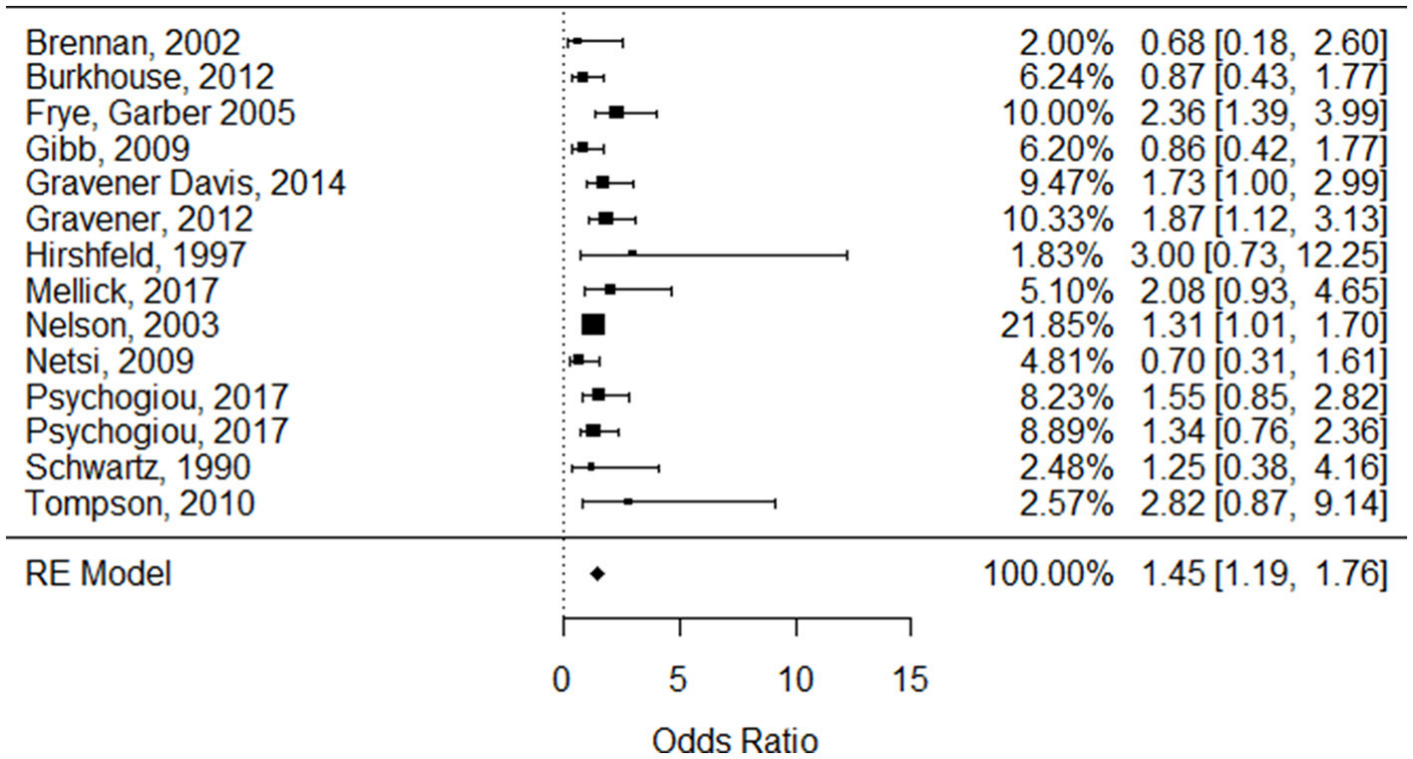
Forest plot for the odds ratio based on the log scale of the association between parental psychopathology and high Expressed Emotion derived from 13 studies (based on 14 independent samples).

Two samples of one study (91) were identified as outliers by using externally standardized residuals. Controlling for those samples did result in marked differences; thus those samples were excluded from further analysis. Reiterating the analysis for the reduced sample under the REM revealed a small effect (92) between parental mental illness and CRIT (${\hat{\mu }}_{0}$ =1.45 [95% CI = 1.19–1.76] *p* <0.001) (see Table 3, Figure 3).

**Table 3:** Summary statistics regarding parental psychopathology and CRIT.

**Study sample**	**Mean ES (OR)**	**95% CI**	***z* score**	**Q**	**τ^2^**	***I* ^2^**	***k***	***N***
Initial sample	1.34	[1.01–1.77]	2.06	35.28**	0.15	57.49	16	2,815
Reduced sample	1.45***	[1.19–1.76]	3.71	16.58	0.02	21.58	14	2,686

*Initial sample, all studies included in analysis; Reduced sample, sample after outlier removal; CI, confidence Interval; Q, Homogeneity statistic; τ^2^, variance between true effects; I^2^ amount of true variance among total variance; k, number of comparison- controls; N, total number of participants.*

*p < 0.05; **p < 0.01; ***p < 0.001.

With the reduced sample, we tested the data under the FEM. The common true effect of the included studies is estimated to be $\hat{\theta }$ = 1.43 [95% CI = 1.23–1.68], *z* = 4.51, *p* < 0.0001). These findings are almost identical to those obtained applying the REM, and results seem to be robust for choosing a meta-analytic model.

### Publication Bias

**Figure 4:**
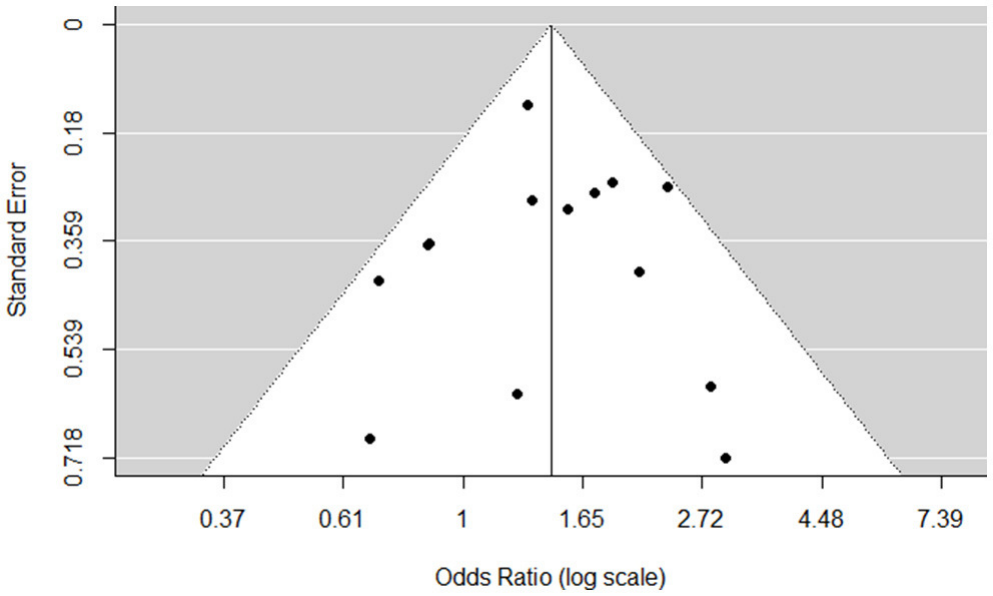
Funnel plot for the odds of the association between parental mental illness and CRIT after removal of outliers.

In terms of potential publication bias, a funnel plot inspection revealed no asymmetrical distribution of the observed effects around the average true effect (see Figure 4). The visual inspection is supported by the rank correlation test (Kendall's τ = −0.09, *p* = 0.67) and the regression test (*z* = −0.15, *p* = 0.88) indicating no funnel plot asymmetry.

Performing Cochrane's *Q*-Test with the reduced sample, heterogeneity remained insignificant (Q = 16.58, df = 13, *p* = 0.22). The variance of the true effect is estimated to be τ^2^ = 0.0277 (SE = 0.0514). The amount of total variability between the observed effect sizes due to heterogeneity is estimated to be *I*^2^ = 21.58%, and was overall low (93). Nevertheless, the moderator analysis seemed appropriate due to the low sample numbers (k) included (see Table 4).

**Table 4:** Results of meta-regression including hypothesized moderators.

**Moderator**	** ${\hat{\beta }}_{i}$ **	**SE**	**95% CI**
Intercept	1.83**	0.67	[0.51, 0.316]
Infant	−1.31	0.70	[−2.68, 0.06]
Pre-schooler	−1.19	0.72	[−2.6, 0.23]
School age	−1.06	0.65	[−2.33, 0.21]
Adolescent	−1.50*	0.68	[−2.83, −0.16]
Child diagnosis	−0.80*	0.31	[−1.40, −0.18]
Male gender	−0.54	2.9	[−1.10, 0.01]

*${\hat{\beta }}_{i}$, estimate of regression parameters; SE, adjusted standard error of regression parameter; CI, confidence interval.*

p < 0.1; *p < 0.05; **p < 0.01; ***p < 0.001.

### Meta-Regression

We performed the meta-regression with the reduced set of samples. Moderator analysis revealed child age as not significant when entered as a continuous variable (${\hat{\beta }}_{i}$ = −0.001, Q_Model_ = 0.002, df = 1, *p* = 0.96). Child age was a significant moderator for the strength of the association between parental mental illness and Crit, when entered as a categorical variable into the model (infants: ${\hat{\beta }}_{i}$ = 0.40,Q_Model_ = 12.36, df = 2, *p* = 0.0021; pre-schoolers: ${\hat{\beta }}_{i}$ = 0.36, Q_Model_ = 12.23, df = 2, *p* = 0.0022; school-age children: ${\hat{\beta }}_{i}$ = 0.36, Q_Model_ = 12.25, df = 2, *p* = 0.0022; adolescents: ${\hat{\beta }}_{i}$ = 0.30, Q_Model_ = 12.30, df = 2, *p* = 0.0021). In accordance with those results, the absence of a child's diagnosis was a significant moderator when analysed as a dichotomous moderator (${\hat{\beta }}_{i}$ = 0.43, *p* < 0.0001, Q_Model_ = 1.86, df = 2, *p* = 0.17) as τ^2^ was reduced, thus explaining the variance through the moderator.

Study quality proved to be a non-significant moderator (${\hat{\beta }}_{i}$ = −0.06, *p* = 0.31) (Q_Model_ = 1.03, df = 1, *p* = 0.31), as did parental diagnostic category (Q_Model_ = 1.02, df = 2, *p* = 0.60). When parental sex was examined (Q_Model_ = 2.49, df = 1, *p* = 0.11), we observed significant associations with the female (${\hat{\beta }}_{i}$ = 0.43, *p* < 0.0001) but not male sex (${\hat{\beta }}_{i}$ = −0.42, *p* = 0.11).

When entering parental sex, presence of the child's diagnosis and child-age category as predictors into the regression model (see Table 4) (Q_Model_ = 11.87, df = 6, *p* = 0.06), the amount of unaccounted variability decreased (*I*^2^ = 0%) and the moderators accounted for *R*^2^ = 100 % of the heterogeneity. The child-age category adolescence (${\hat{\beta }}_{i}$ = −1.495, *p* = 0.02) and presence of the child's diagnosis (${\hat{\beta }}_{i}$ = −0.80, *p* = 0.01) remained significant predictors in the multiple meta-regression with adolescents producing small effects ($\hat{\mu }$ = 1.40) and children with a diagnosis producing medium effects ($\hat{\mu }$ = 2.82) (92) on EE when living with a parent suffering from a mental illness.

## Discussion

The aim of the present meta-analysis was to estimate the overall effects of a parental mental illness on EE compared to controls without any mental illness within the literature. With respect to existing studies on EE and youth psychopathology, we were able to depict a small but significant overall effect (OR = 1.45) between parental mental illness and CRIT. This finding provides support for our assumption that parental CRIT is a specific reactional style of parents with a mental illness and more frequent in parent-child relations within their families. Parents with a mental illness tend to react more critically and make more critical statements when asked about their relationship with their child. For this reason, HEE cannot be regarded as only a reaction to children's psychopathology (44), but as an interactional style in families in which a parent has a mental illness. The existence of a critical, negative family climate and harsh, intrusive parenting behavior thus proves to be a robust risk factor for a child's socio-emotional development (94), and as a stressor that may interact with a child's vulnerability (39) and stress reactivity (95). Thus, parental criticism may act as one mechanism in the transgenerational transmission of mental illness (9, 10). This effect proved to be stable toward the choice of the meta-analytic model and without significant publication bias.

Our analysis was limited to the coding of CRIT and lacks information on EOI, because data on EOI was neither sufficiently available nor reported, and the present studies mainly reported on CRIT. This is not very surprising as the use of EOI in studies on children lacks validity and is under discussion (24). An adaption of the EOI construct has already been demanded elsewhere and suggestions have been made to only incorporate self-sacrifice and overprotection, as those appear developmentally salient. Statements of attitude, excessive detail, and emotional displays within EOI do not appear striking when made by a parent about a minor child (24, 25, 96).

Surprisingly, only one (91) out of 13 studies used the CFI to assess EE. There appears to be a trend in studies published after 1997 using the shorter FMSS rather than the CFI, which initially was considered as gold standard tool to assess EE (21, 26).

Unfortunately, our sample only consisted of parents with depression and anxiety disorders, and our findings are limited to that diagnosis spectrum. CRIT can be regarded as reflecting the attributional and cognitive style typical of depression (97). However, information on EE in the families of parents with mental disorders other than depression and anxiety is urgently required to improve our understanding of family interactions, especially EE, as a mechanism of transmission.

Our sample consisted predominantly of mothers with a mental illness, and female controls. Unfortunately, 1,533 dyads could not be allocated to the clinical or control group due to correlational data from the studies included. Future studies should aim for a more balanced sex relation and be clear about group allocation. Female sex functioned as a significant moderator. Nonetheless, we cannot draw any conclusions about fathers and CRIT based on our data. This finding is congruent with the literature, because fathers have been neglected in the research on parents with a mental illness (98). There are indications that fathers with depression do not present with higher levels of HEE or CRIT, but that they do make fewer warm, positive remarks than healthy controls (36). This indicates a potential sex difference in the reactional and interactional style of parents with a mental illness, but it is a difference that needs clarification. The presence or absence of paternal warmth should come to the fore when studying fathers with a mental illness in the future, because that factor is not automatically included in the HEE/CRIT code and only is depicted indirectly within the LEE rating as it is one component that is rated and conglomerated into LEE/HEE. Parental sensitivity and warmth appear to be strong behavioral protective factors for children's development and pathology in the preschool age in the Transgenerational Transmission of Mental Disorders (99). Based on this consideration, a sex difference in the EE of preschoolers' parents and especially of the positive component warmth, is particularly important. Future studies should consider to report the level of parental warmth in addition to the HEE/LEE rating.

Implementing adolescent age into the regression resulted in small effects (OR = 1.40), but we can make no statement about younger ages. We were able to show a significant increase in overall effects (OR = 2.82) when children were presenting with a mental health problem themselves, providing support for EE CRIT acting as a mechanism in the transgenerational transmission of mental disorders. Considering HEE's prognostic power in predicting treatment response in adolescents (61–63), this finding appears fundamental. Adolescents with eating disorders show worse treatment response when living with a HEE parent. But the parental attitude about the relationship to the child does not only seem to be influenced by the burden provoked by the child's mental illness but the parent's mental health as well. Parents with a mental illness make more CRIT statements than healthy controls. These results support that children of parents with a mental illness are exposed to more CRIT in their home environment and, as they develop symptoms themselves, face even more parental CRIT and therefore are exposed to greater challenges in responding to treatment. Assessing parental psychopathology should be implemented in future studies observing EE and child treatment response.

One additional possible explanation is that genetically vulnerable children who may have a difficult temperament are exposed to overly critical parents, develop problem behavior and psychopathology over time. The children's problem behaviors provoke even more negativity and criticism from already burdened parents, leading to an internal vicious circle of mental illness, critical cognitions, attitudes, and reactions the children might adapt while growing up that appear on the level of family interactions in the Transgenerational Transmission of Mental Disorders system.

To our knowledge, this meta-analysis is the first to assess the overall effects of the presence or absence of a parental mental illness on CRIT, and to integrate the concept of CRIT within the Transgenerational Transmission of Mental Disorders system. It is important to identify CRIT's wider prevalence in parents with a mental illness, because future therapeutic interventions may identify and target parental CRIT as a specific component of parent-child-relations and reflection of the family climate in clinical assessments. As behavioral observations of parent-child-interactions are so time-consuming, costly, and require extensive training of observers, EE carries the potential to detect disrupted intra-family interactions within families of parents with a mental illness in everyday therapeutic interventions.

### Limitations

We were unable to differentiate the children's mental illnesses, nor whether they were suffering from either internalizing or externalizing disorders. There was also a lack of specific information on children's age in the studies included. It is important to clearly differentiate children's age, and not just age categories, because the exposure to CRIT at an early age predicts the development of problem behavior later in life (38). This is essential, as during the first 3 years of life, children are especially vulnerable to dysfunctional, insensitive parent-child-interactions (100, 101) and the risk for psychopathology in offspring rises when a child is exposed to a stressful, critical home environment (51) and HEE parental attitudes. In future studies it would be worthwhile to focus on particularly vulnerable ages and insensitive parenting, in particular CRIT, to be able to adapt and implement preventive programs at an early stage.

Study quality did not function as a moderator, and inter-rater-agreement was medium despite the extensive training of coders. Furthermore, inter-rater agreement of the study variable *type of comparison* was moderate due to the difficulty of rating comparisons in population-based studies. Our search was restricted to articles in English and German, which may have precluded the identification of other relevant studies, although we included the gray literature to avoid the “file drawer-problem”, as published studies most often report significant findings that disturb the overall balance of results. Furthermore, data was exclusively descended from English speaking countries within the Organization for Economic Co-operation and Development (OECD).

The systematic literature search was updated last in November 2021, thus potentially new articles published after November 2021 are not included in this review. However, in order to be able to complete a review and meta-analysis, one has to come to a decision of when to stop and we believe that we were able to arrive at significant results with the studies included, especially in light of the fact that results of the publication bias analysis do not indicate a distortion and according to fail safe n analysis k = 36 studies would need to be included to change the result to non-significance.

## Conclusions

The current study highlights the dearth of studies on EE in families of mentally ill parents and their children, who already carry a higher risk of developing mental illnesses themselves. Established effects of CRIT and parental mental illness are significant, although generally small, and become stronger as offspring develop mental disorders themselves. These results support the importance of HEE/CRIT as a mechanism in the Transgenerational Transmission of Mental Disorders and as a firm component of dysfunctional parent-child-interactions. Future studies are needed to deepen our understanding of EE and particularly of EOI and warmth in families in which parents suffer from a mental illness. The research on EE in families of children of parents with a mental illness has the potential to guide future preventive interventions and may be exploited in interventions especially developed to improve parent-child-relations.

## Data Availability Statement

The original contributions presented in the study are included in the article/Supplementary Materials, further inquiries can be directed to the corresponding author/s.

## Author Contributions

JF designed the study, performed and updated the search strategy, completed the statistical analysis, and wrote the protocol. LMD did the independent full text screening as second rater and was involved in the inclusion process of studies. NB did the quality assessment and coding of study variables as second rater. JA gave methodological advice and supported the drafting of the manuscript. HC functions as PHD advisor and supervised the study and preparation of manuscript and commented on the whole manuscript. All authors contributed to the article and approved the submitted version.

## Conflict of Interest

The authors declare that the research was conducted in the absence of any commercial or financial relationships that could be construed as a potential conflict of interest.

## Publisher's Note

All claims expressed in this article are solely those of the authors and do not necessarily represent those of their affiliated organizations, or those of the publisher, the editors and the reviewers. Any product that may be evaluated in this article, or claim that may be made by its manufacturer, is not guaranteed or endorsed by the publisher.

## Figures and Tables

**Table A1:** Full search strategy for databases; terms within categories were combined with OR, between categories with AND.

**Population**	**Disorder**	**Intervention**
• Parent* • Child • Maternal • Parental • “Mentally ill parents”	• Mental* • Mental illness • Mentally ill • Mental disorder* • Mental health problems • Mental disease • Affective disorder* • Bipolar disorder • Anxiety disorder • Phobia • Specific phobia • Generalized anxiety disorder • Depression* • Panic disorder* • Substance abuse • Addictive disorder • Addiction* • Eating disorder* • Anorexia nervosa • Bulimia nervosa • Insomnia • Obsessive compulsive disorder* (OCD) • Impulse control disorder* • Adjustment disorder* • Post traumatic stress disorder* (PTBS) • Personality disorder* • Borderline • Somatoform disorder* • Hypochondria • Psychososmatic disorder*	• Expressed Emotion • EE • Five minute speech sample • Camberwell family interview • Family questionnaire • Family attitude scale

*truncation or wildcard.

## Footnotes

^1^Mental illness as diagnosed by a validated, standardized clinical assessment tool (e.g., clinical interview).
