# Supplementary material for: Expressed Emotion in the Family: A Meta-Analytic Review of Expressed Emotion as a Mechanism of the Transgenerational Transmission of Mental Disorders
Source: Front Psychiatry. 2022 Feb 1;12:721796. doi: 10.3389/fpsyt.2021.721796 (PMC8846301; doi:10.3389/fpsyt.2021.721796)
Supplement: Supplementary file 1 [file Table_1.DOCX]

Rating of study quality according to tot he Newcastle Ottawa Scale (NOS)

| Study | Selection | | | | Comparability | | | Exposure | | | |  | |  | |  |
| --- | --- | --- | --- | --- | --- | --- | --- | --- | --- | --- | --- | --- | --- | --- | --- | --- |
|  | Item 1 | Item 2 | Item 3 | Item 4 | | Item 5a | Item 5b | | Item 6 | Item 7 | Item 8 | | Quality Score NB | | Quality Score  JF | |
| Brennan, Hammen, Katz, & Le Brocque (94) | 1 | 1 | 1 | 1 | | 1 | 1 | | 1 | 1 | 1 | | 9 | | 8 | |
| Burkhouse et al. (95) | 1 | 1 | 1 | 1 | | 1 | 1 | | 1 | 1 | 0 | | 8 | | 7 | |
| Frye & Garber (43) | 1 | 1 | 1 | 1 | | 0 | 0 | | 0 | 1 | 1 | | 6 | | 7 | |
| Gibb et al. (96) | 1 | 1 | 1 | 1 | | 0 |  | | 1 | 1 | 1 | | 7 | | 6 | |
| Gravener Davis (97) | 1 | 0 | 0 | 1 | | 1 | 1 | | 1 | 1 | N.A. | | 6 | | 4 | |
| Gravener et al. (35) | 1 | 0 | 0 | 1 | | 1 | 1 | | 1 | 1 | N.A. | | 6 | | 8 | |
| Hirshfeld et al. (98) | 1 | 0 | 1 | 1 | | 1 | 1 | | 1 | 1 | 0 | | 7 | | 8 | |
| Mellick et al. (99) | 1 | 0 | 0 | 0 | | 1 | 1 | | 1 | 1 | N.A. | | 5 | | 6 | |
| Nelson et al. (100) | 1 | 1 | 1 | 1 | | 1 | 1 | | 1 | 1 | 1 | | 9 | | 9 | |
| Netsi (37) | 1 | 1 | 1 | 1 | | 1 | 1 | | 0 | 1 | 1 | | 8 | | 7 | |
| Psychogiou (101) | 1 | 1 | 1 | 1 | | 1 | 1 | | 0 | 1 | 1 | | 8 | | 9 | |
| Schwartz et al. (83) | 1 | 0 | 0 | 0 | | 0 | 0 | | 1 | 1 | N.A. | | 3 | | 4 | |
| Tompson et al. (102) | 1 | 0 | 0 | 0 | | 1 | 1 | | 1 | 1 | N.A. | | 5 | | 0 | |

Annotation: Item ratings refer to ratings of rater NB.; N.A. = Not applicable.
